# Endoscopic ultrasound-guided drainage of a prevertebral neck abscess: a case report

**DOI:** 10.1055/a-2665-7534

**Published:** 2025-08-20

**Authors:** Haojie He, Zecan Shi, Weigang Gu, Lei Lu, Jianfeng Yang, Xiaofeng Zhang, Hangbin Jin

**Affiliations:** 1 The Fourth School of Clinical Medicine, Zhejiang Chinese Medical University, Hangzhou First Peopleʼs Hospital, Hangzhou, Zhejiang, China; 2 Department of Gastroenterology, Affiliated Hangzhou First People’s Hospital, School of Medicine, Westlake University, Zhejiang, China; 3 Key Laboratory of Integrated Traditional Chinese and Western Medicine for Biliary and Pancreatic Diseases of Zhejiang Province, Hangzhou, China; 4 Hangzhou Institute of Digestive Diseases, Zhejiang, China

A 73-year-old male presented with a 20-day recurrent fever (peak 40.2°C) without any symptoms. Laboratory findings showed neutrophilia (7.2 × 10 ^9^ /L, 82.8%), elevated CRP (219 mg/L) and PCT (0.83 ng/mL), and blood cultures positive for *Klebsiella pneumoniae* bacteremia, prompting meropenem therapy. Subsequent posterior neck pain and progressive upper limb weakness prompted cervical magnetic resonance imaging (MRI), revealing a 32 mm × 19 mm × 29 mm prevertebral abscess anterior to C4 ( Fig. 1 ). Due to its deep location and proximity to neurovascular structures, conventional percutaneous ultrasound or computed tomography (CT)-guided drainage was deemed high-risk. A multidisciplinary team (MDT) opted for endoscopic ultrasound (EUS)-guided drainage. EUS identified a 32.5 mm × 25.9 mm hypoechoic mass with patchy hyperechoic areas in the posterior hypopharyngeal wall. The abscess was punctured with a 19-G needle, and reddish fluid was aspirated ( Fig. 2 ). A guidewire was inserted into the abscess cavity. After dilation by using a cystotome, a drainage tube was placed ( Video 1 ). The follow-up CT scan revealed the drainage tube in an optimal position ( Fig. 3 ). Pus culture confirmed *K. pneumoniae* . However, nonliquefaction of the abscess resulted in limited fluid drainage. Thereafter, the patient was transferred to orthopedic surgery.

**Fig. 1:**
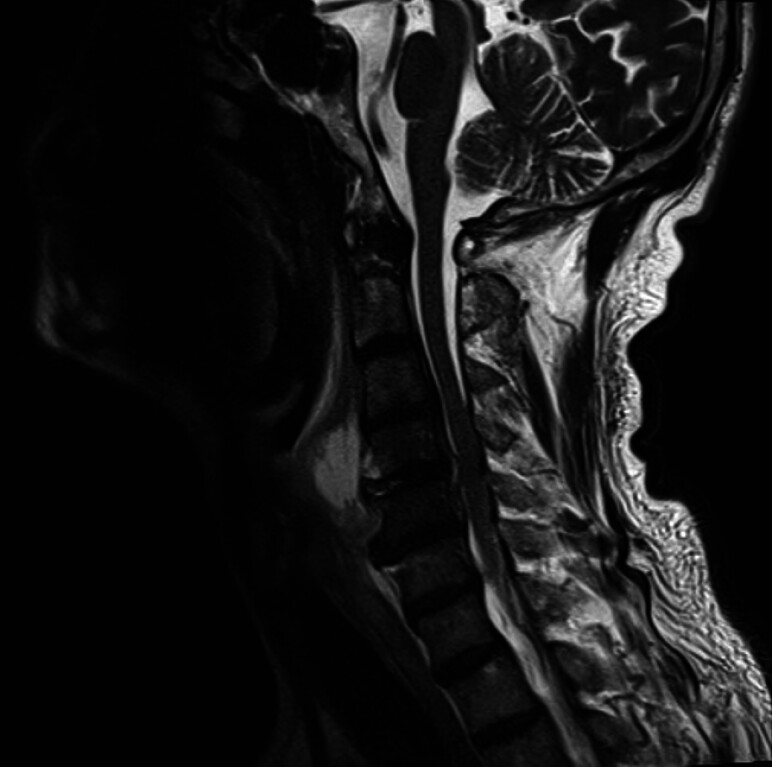
Cervical MRI revealing a prevertebral abscess at C4.

**Fig. 2:**
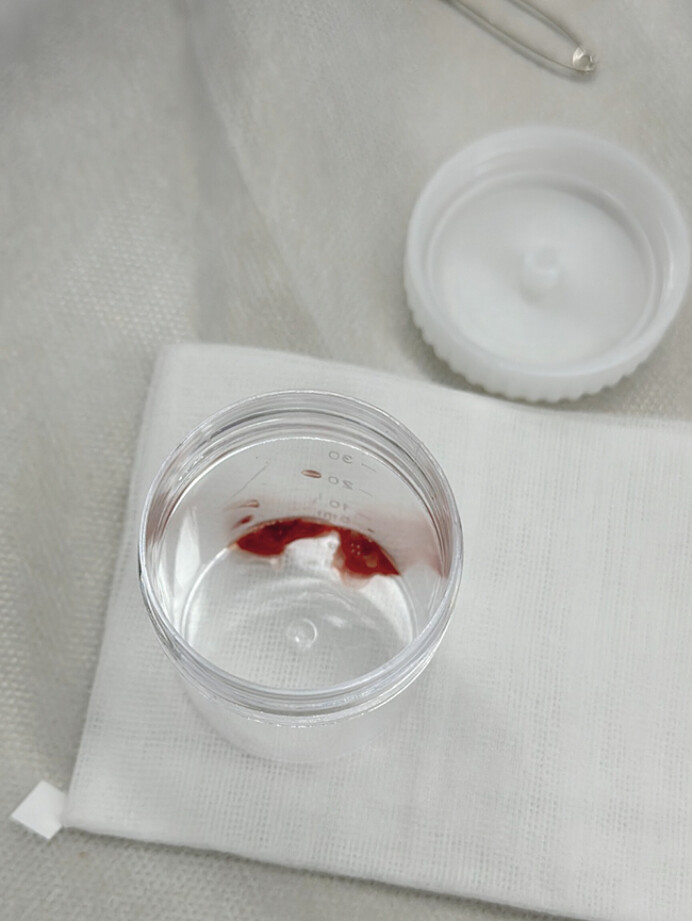
Reddish fluid aspirated from the abscess.

**Fig. 3:**
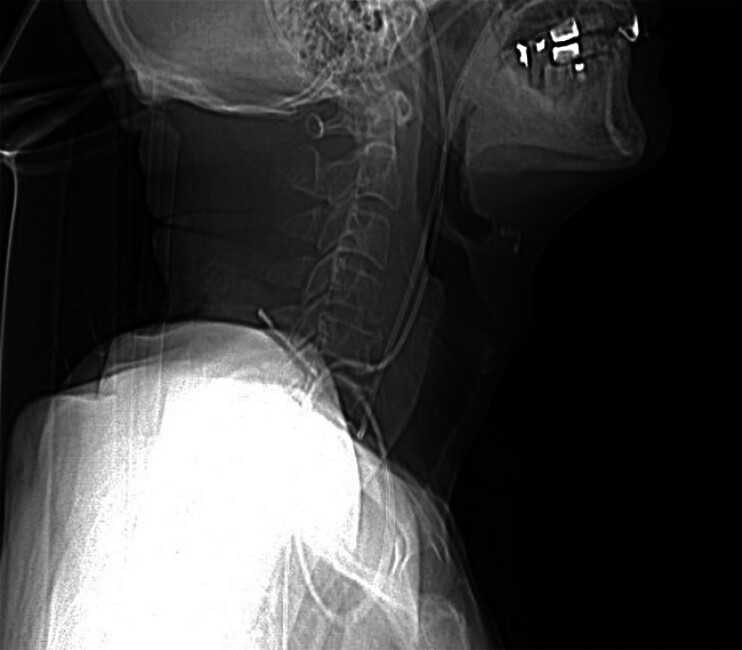
The follow-up CT scan revealing the drainage tube in an optimal position.

EUS-guided drainage of a prevertebral neck abscess.Video 1

The prevertebral space, extending from the skull base to coccyx, is prone to abscess formation secondary to spinal degeneration, infection, or trauma, necessitating early drainage to prevent spinal cord compression 1 . While ultrasound-guided drainage matches surgical efficacy with lower invasiveness 2 , EUS offers distinct advantages: trans-luminal access via natural orifices shortens puncture distance, enables real-time needle visualization (reducing neurovascular injury risk), and avoids skin puncture (minimizing exogenous infection) 3 4 . This represents the first reported EUS-guided drainage of a prevertebral abscess, highlighting its utility for deep-seated lesions adjacent to the digestive tract.

Endoscopy_UCTN_Code_TTT_1AS_2AB

## Notes

Endoscopy E-Videos https://eref.thieme.de/e-videos
*E-Videos* is an open access online section of the journal *Endoscopy* , reporting on interesting cases and new techniques in gastroenterological endoscopy. All papers include a high-quality video and are published with a Creative Commons CC-BY license. Endoscopy E-Videos qualify for HINARI discounts and waivers and eligibility is automatically checked during the submission process. We grant 100% waivers to articles whose corresponding authors are based in Group A countries and 50% waivers to those who are based in Group B countries as classified by Research4Life (see: https://www.research4life.org/access/eligibility/ ). This section has its own submission website at https://mc.manuscriptcentral.com/e-videos .
